# Genomic characterization of an emerging Enterobacteriaceae species: the first case of co-infection with a typical pathogen in a human patient

**DOI:** 10.1186/s12864-020-6720-z

**Published:** 2020-04-15

**Authors:** Zhao Zhang, Daixi Li, Xing Shi, Yao Zhai, Yatao Guo, Yali Zheng, Lili Zhao, Yukun He, Yusheng Chen, Zhanwei Wang, Jianrong Su, Yu Kang, Zhancheng Gao

**Affiliations:** 1 Department of Respiratory & Critical Care Medicine, Peking University People’s Hospital, Beijing, Beijing China; 2 Department of Respiratory & Critical Care Medicine, the First Affiliated Hospital of Zhengzhou University, Zhengzhou, Henan China; 3 Department of Respiratory and Critical Care Medicine, Zhongshan Hospital Xiamen University, Xiamen, 361004 Fujian China; 4 Beijing Institute of Genomics, Chinese Academy of Sciences, Beijing, Beijing China; 5 University of Technology Sydney, Ultimo, NSW Australia; 6 Department of Respiratory, Critical Care and Sleep Medicine, Xiamen University Xiang‘an Hospital, Xiamen, Fujian China; 7 Department of Respiratory & Critical Care Medicine, Fujian Provincial Hospital, Fuzhou, Fujian China; 8 Laboratory Medicine, Peking University People’s Hospital, Beijing, China; 9 Department of Clinical Laboratory Center, Beijing Friendship Hospital, Beijing, Beijing China

**Keywords:** Enterobacteriaceae, Pathogen, Whole-genome sequencing, RNA-Seq, Phylogenetic

## Abstract

**Background:**

Opportunistic pathogens are important for clinical practice as they often cause antibiotic-resistant infections. However, little is documented for many emerging opportunistic pathogens and their biological characteristics. Here, we isolated a strain of extended-spectrum β-lactamase-producing *Enterobacteriaceae* from a patient with a biliary tract infection. We explored the biological and genomic characteristics of this strain to provide new evidence and detailed information for opportunistic pathogens about the co-infection they may cause.

**Results:**

The isolate grew very slowly but conferred strong protection for the co-infected cephalosporin-sensitive *Klebsiella pneumoniae*. As the initial laboratory testing failed to identify the taxonomy of the strain, great perplexity was caused in the etiological diagnosis and anti-infection treatment for the patient. Rigorous sequencing efforts achieved the complete genome sequence of the isolate which we designated as AF18. AF18 is phylogenetically close to a few strains isolated from soil, clinical sewage, and patients, forming a novel species together, while the taxonomic nomenclature of which is still under discussion. And this is the first report of human infection of this novel species. Like its relatives, AF18 harbors many genes related to cell mobility, various genes adaptive to both the natural environment and animal host, over 30 mobile genetic elements, and a plasmid bearing *bla*_CTX-M-3_ gene, indicating its ability to disseminate antimicrobial-resistant genes from the natural environment to patients. Transcriptome sequencing identified two sRNAs that critically regulate the growth rate of AF18, which could serve as targets for novel antimicrobial strategies.

**Conclusions:**

Our findings imply that AF18 and its species are not only infection-relevant but also potential disseminators of antibiotic resistance genes, which highlights the need for continuous monitoring for this novel species and efforts to develop treatment strategies.

## Background

Antimicrobial resistance (AMR) is an increasingly global health threat that contributes to 700,000 deaths per year [1]. Increased and often unrestricted antibiotic use in the clinical and farming settings is to blame for this issue. Growing surveillances based on genomic sequencing of microbes from the natural environment, human settlements, and clinical settings have been conducted worldwide to investigate the evolution and transfer of antibiotic resistance genes (ARGs) [2–4]. In recent years, the eco-evolutionary feedback loops between ecological and evolutionary dynamics have been increasingly recognized, where spillover of antibiotic use to natural and semi-natural environments may have profound implications on the distribution of ARGs in natural bacterial populations which serve as environmental reservoirs of resistance determinants [5, 6]. However, how resistance evolves, and how ARGs are maintained and dispersed back to clinical settings is poorly understood. Understanding the dynamics of the continuous feedback loops from clinical to nature and back may prove critical for preventing and controlling the problem of antibiotic resistance.

The rapidly developing sequencing technology increasingly enables the identification of emerging opportunistic pathogens and taxonomical classification based on their genomic information [7–9]. Naturally, opportunistic pathogens inhabit in the natural environment and are occasionally resistant to common antibiotics. Among these previously unknown pathogens, many are belong to species of the *Enterobacteriaceae* family [10, 11]. Meanwhile, many *Enterobacteriaceae* species are commensal microbiota of human and animal guts, but under certain conditions, can be opportunistic pathogens that cause infections [12]. These species often have other animal hosts, or they can be found in more diverse environments, such as soil and sewage [13]. *Enterobacteriaceae* species (including *E. coli*, *Klebsiella*, and *Enterobacter*) are also famous for their antibiotic resistance and regarded as some of the most dangerous pathogens since they can efficiently acquire various ARGs through efficient plasmid transmission [14]. The ability of these species to disseminate between habitats and transferring ARGs highlights their importance as mediators in the eco-evolutionary feedback loops that disperse ARGs from natural environments back to clinical settings. The taxonomy of *Enterobacteriaceae* is complex, containing 28 genera and over 75 species [15], while novel species are continuously discovered. Recognizing and characterizing *Enterobacteriaceae* species, especially those of emerging opportunistic pathogens, is critical for understanding the dynamics of the evolution of AMR.

Here, we isolated from a patient with a biliary infection a novel strain of unknown taxonomy accompanying an infectious *Klebsiella pneumoniae* strain, which we designated as AF18. AF18 grew slowly but provided drug-resistance to its companion by carrying a *bla*_CTX-M-3_ resistant gene. The co-infection brought perplexity in both diagnosis and treatment of the patients. In order to provide new evidence and detailed information for opportunistic pathogens about the complex issues that they may cause in clinical infections, we conducted a study with the three following objectives: (1) Clarifying the taxonomy of AF18 using whole-genome phylogenetic analysis; (2) Testing the ability of AF18 to protect *K. pneumoniae* from antibiotics in co-culture experiments; and (3) Analyzing the adaptation mechanisms of AF18 base on transcriptome sequencing. Finally, we find that AF18 is a strain of an undefined novel species in the family *Enterobacteriaceae*, and that sensitive *K. pneumoniae* can survive when co-cultured with AF18 in Luria-Bertani broth containing 8 μg/mL ceftriaxone. Furthermore, genomic and transcriptomic analyses reveal the genomic characteristics of this rare pathogen and the regulation mechanisms of how it adapts to multiple habitats and its association with ARGs transfer.

## Results

### Biological identification of the strain AF18

**Fig. 1:**
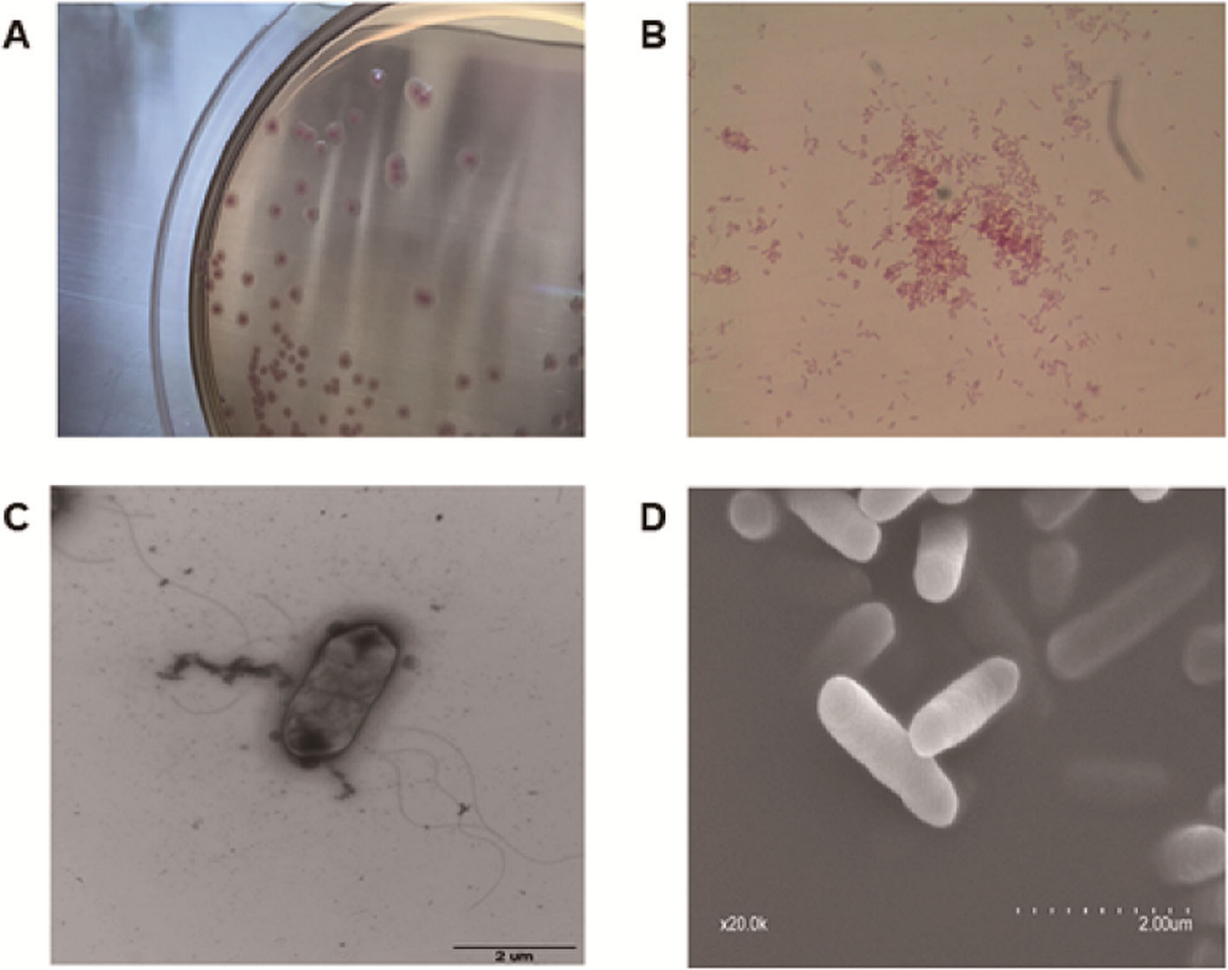
The morphological characters of AF18. **a** The morphology of AF18 colonies on MacConkey agar plate. **b** Gram staining of AF18 cells. **c** Flagella of AF18 photographed by transmission electron microscopy. **d** Cells of AF18 under scanning electron microscopy

From the bile sample of the patient, two types of colonies were isolated after serial dilutions and isolations on MacConkey agar plates. One type was mucous, entirely pink, and of 4-5 mm in diameter, which was finally identified as a *K. pneumoniae* clone sensitive to common antibiotics (Table 1); the other type was composed by small (2-3 mm in diameter) red-centered colonies with clear and transparent edges (Fig. 1a). The bacteria of the small colonies seemed prone to adhere to the cells of *K. pneumoniae* and were not able to be isolated until extensive dilutions. The taxonomy of the small colonies was not immediately identified by the microbiological laboratory in the hospital, and we designated it as strain AF18. AF18 exhibited resistance to most β-lactam antibiotics in antimicrobial susceptibility testing (Table 1). As the infection was rather intractable and finally cured by intravenous amikacin, the final diagnosis for the patient was a co-infection caused by a sensitive *K. pneumoniae* strain and a multidrug-resistant strain of unknown species.

**Table 1:** The antibiotic resistance profile of AF18 and *K. pneumoniae* isolate

Drug	Antibiotic susceptibility			
	AF18		*K. pneumoniae* strain	
	MIC (μg/ml)	Phenotype	MIC (μg/ml)	Phenotype
Ampicillin	≥32	R	16	I
Ampicillin/sulbactam	≥32	R	4	S
Piperacillin	≥128	R	≤4	S
Piperacillin/tazobactam	≥128	R	≤4	S
Cefazolin	≥64	R	≤4	S
Cefuroxime	≥64	R	≤1	S
Cefuroxime axetil	≥64	R	≤1	S
Cefotetan	≤4	S	≤4	S
Ceftazidime	16	R	≤1	S
Ceftriaxone	≥64	R	≤1	S
Cefepime	≥64	R	≤1	S
Aztreonam	≥64	R	≤1	S
Imipenem	≤1	S	≤1	S
Meropenem	≤0.25	S	≤0.25	S
Amikacin	≤2	S	≤2	S
Gentamicin	≤1	S	≤1	S
Tobramycin	2	S	≤1	S
Ciprofloxacin	2	I	≤0.25	S
Levofloxacin	1	S	≤0.25	S
Nitrofurantoin	256	R	≤16	S
Trimethoprim/sulfamt	≤20	S	≤20	S

Microscope observation showed that AF18 was a Gram-negative bacillus (Fig. 1b), and its cells were surrounded by flagella under a transmission electron microscope (Fig. 1c). The scanning electron microscope confirmed the tubular shape of AF18 and a smooth surface with no polysaccharide particles (Fig. 1d), in line with the mucus-free characteristics of its colonies. VITEK-II in the hospital laboratory did not identify any bacterial species with identical biochemical properties to AF18 (Table S1), whereas the API20E biochemical identification system suggested AF18 as *Pantoea* sp. but with low reliability. The mass spectrometry which scans the protein profile of samples did not identify the species of AF18 either.

### Complete genome of Enterobacteriaceae bacterium AF18

To determine the taxonomy and genetic features of AF18, we performed whole-genome sequencing using two platforms, Illumina Hiseq (generates short-reads) and PacBio sequencer (generates long-reads), obtaining a high-quality completed genome sequence. AF18 possessed a circulated chromosome and two plasmids **(**Table 2**).**

**Table 2:** Overview of genome information for AF18

Replicon	Nucleotide length (bp)	Coding Genes	GC%	Inc type	GenBank ID
Chromosome	5,676,372	5651	53.06	NA	CP025982
pAF18_1	140,420	181	51.14	IncFII	CP025983
pAF18_2	42,923	53	51.28	IncN	CP025984

**Fig. 2:**
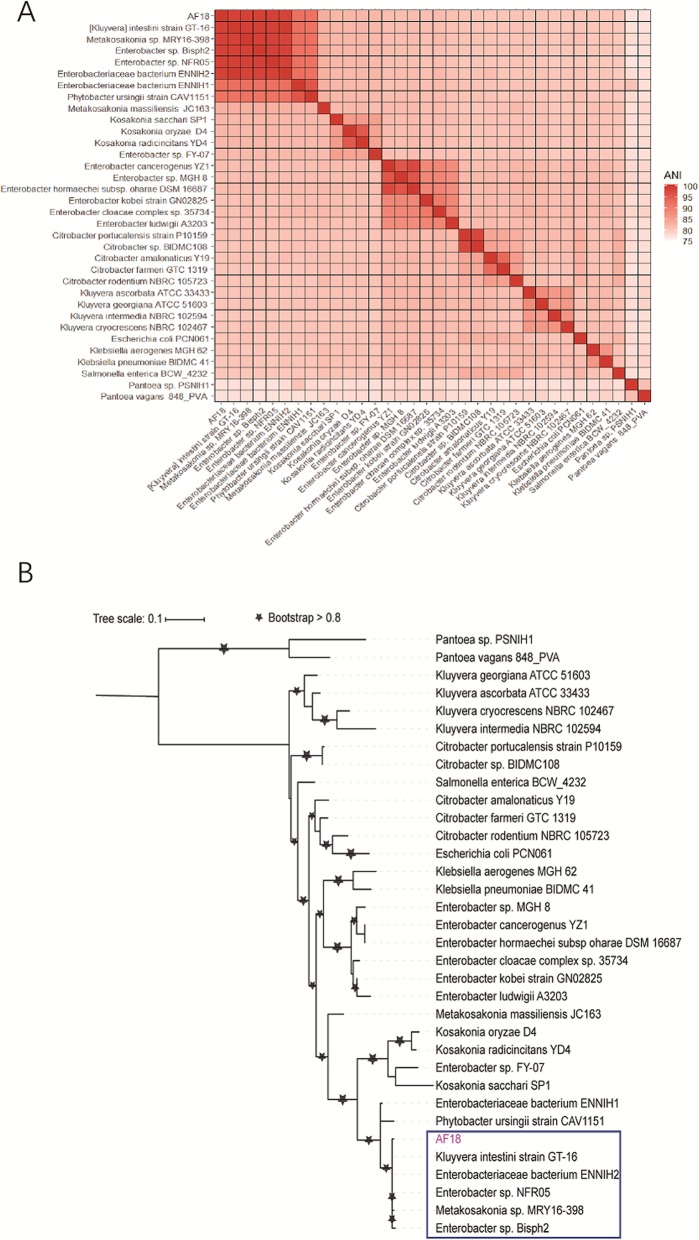
Phylogenetic relationship of 34 strains related to AF18. **a**. The heatmap of ANI matrix. The color bar represents the value of ANI. The top five species (not including AF18) are the closest relatives of AF18 with ANI > 98.5%. **b**. The maximum likelihood phylogenetic tree constructed based on the core genome SNPs. The species in the blue box are the closest relatives of AF18 in the phylogenetic tree which are the same as the top five species of ANI heatmap. ANI, average nucleotide identity

By using Mash [16] to search the publicly available bacterial genomes and drafts with a cutoff of mutation distance < 0.25, we identified 33 non-redundant close relatives of AF18, all of which were in the *Enterobacteriaceae* family (Table S2). The average nucleotide identity (ANI) matrix of the 34 strains (Fig. 2a) shows that the closest five with identity > 98.5% (> 95% regarded as strains of the same species [17]) are nominated as *[Kluyvera] intestini* (GCA_001856865.3), *Metakosakonia sp.*(GCA_003925915.1), *Enterobacter sp.* (GCA_000814915.1, GCA_900168315.1), and just *Enterobacteriaceae bacterium* (GCA_002903045.1). The phylogenetic relationship of these relatives was further inferred with core genome SNPs (Fig. 2b)**,** which confirmed the relationships inferred from the ANI matrix and indicated the novel species, including AF18, possibly represents another genus than *Kluyvera*. Herein, we temporarily nominated our stain as *Enterobacteriaceae bacterium* AF18 as the nomenclature of its genus and species is still undefined.

We predicted seven copies of 16S rDNA sequences in AF18. We aligned them to the 33 genomes we picked using BLASTN and calculated the average identity. We removed the genomes which do not contain high quality 16S rDNA sequence. The result shows a good congruence of 16S rDNA and whole-genome comparisons (Table S3). However, considering cutoffs commonly used for intra-species classification by whole-genome ANI > 95% [17] and 16S rDNA identity > 99% [18], 16S rDNA classification found two more strains of the species, namely *Enterobacteriaceae bacterium* ENNIH1, and *Phytobacter ursingii* strain CAV1151 (Table S3). Thus, we think that 16S rDNA can also be used as a marker gene to clarify the taxonomy of isolated strains, but we need to examine the identity cutoff we used carefully.

The chromosome of AF18 possesses 5651 protein-coding genes whose functions facilitate the survival and adaptation of AF18 in various habits (Table S4, Table S5). For example, motility-related genes, including a complete flagellar gene cluster that encodes all components of flagellar, *csg* gene cluster that encodes curli assembly proteins to mediate adhesion, and other genes of *ompA*, *pilRT*, *ibeB*, *icaA*, *htpB* and *fimB*, together confer the ability of adhesion, invasion, chemotaxis, and escape to the host strain. Efflux pump genes which confer resistance to macrolides, quinolones and aminoglycosides were also identified. Meanwhile, the AF18 genome possesses 20 genomic islands, 11 prophages, and five CRISPR sequences (Table S5), suggesting the active transfer of stress-adaptive genes by these mobile genetic elements in this species. More importantly, markers of soil-inhabiting bacteria, including a complete nitrogen fixation gene cluster and *ksgA*—— a pesticide-resistant gene, were found in AF18 genome, which suggests that AF18 is able to colonize natural environments. The mobility of this strain may potentiate its dissemination to various habits.

**Fig. 3:**
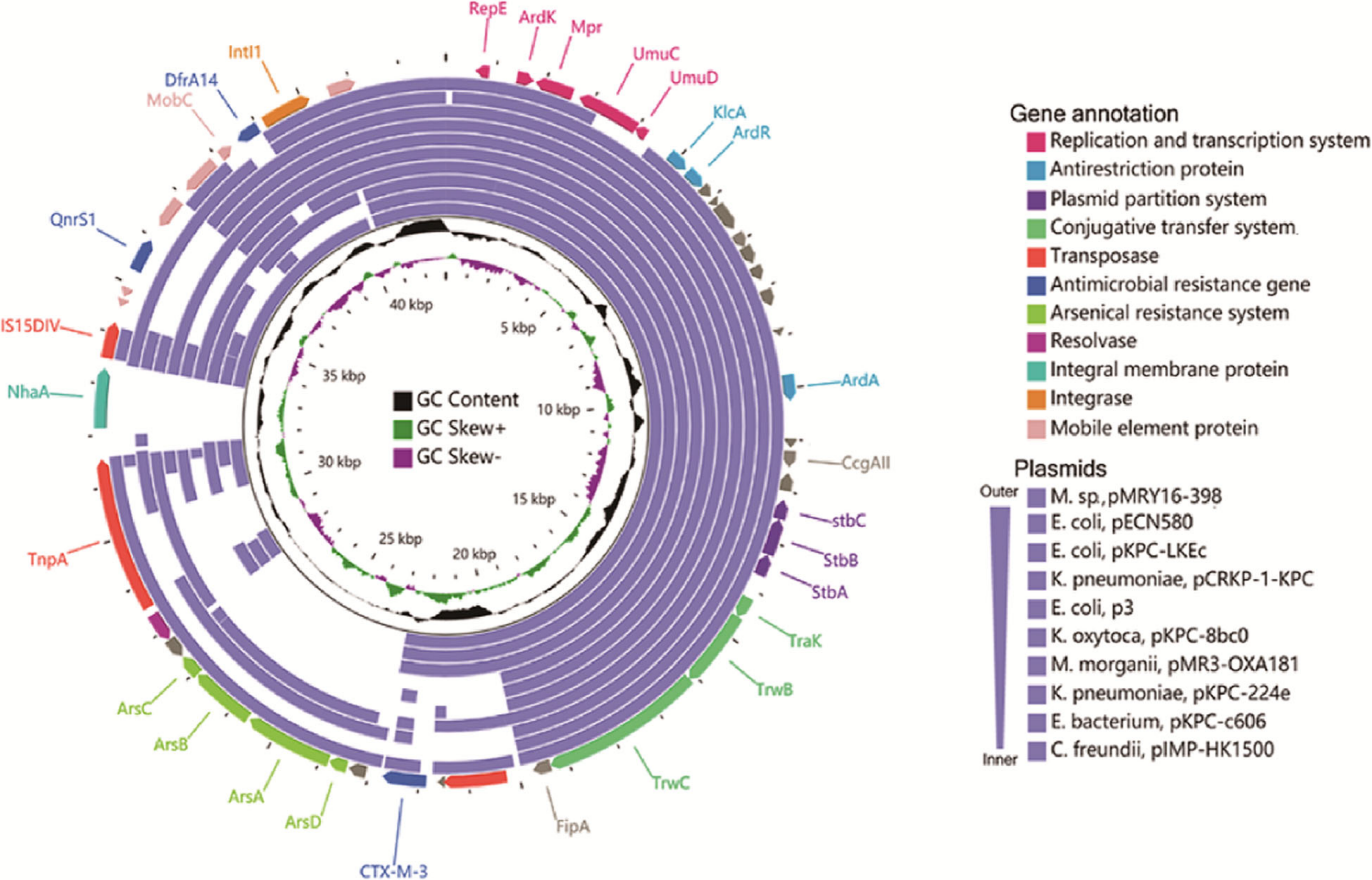
The circular map of pAF18_2 and comparison to similar plasmids. The outmost slot represents the predicted genes of pAF18_2, whose functions are shown in different color arrows. From outward, slot 2–11 indicate aligned fragments from similar plasmids of IncN. Slot 12, GC content; slot 13, GC skew. Accession numbers of plasmids from outer to inner were: AP018758.1, KF914891.1, KC788405.1, KX928750.1, CP028486.1, CP026277.1, KM660724.1, CP026179.1, CP026198.1, KT989599.1

Analysis of conserved genes in plasmids shows that most of the antibiotic-resistant genes of AF18, including *qnrS*, *dfrA*, and *bla*_CTX-M-3_, are carried by the smaller plasmid pAF18_2 (Fig. 3, Table S4) which is, in major part, responsible for the antibiotic resistance profile of AF18 (Table 1). Sequence alignment shows that pAF18_2 is similar to many plasmids from other *Enterobacteriaceae* species, such as *E. coli* (KF914891.1, KC788405.1, CP028486.1), *K. pneumoniae* (KX928750.1, CP026179.1), and *C. freundii* (KT989599.1), and they contain identical replication origins, replication and transcription systems, plasmid partition systems, and a partial gene cluster responsible for plasmid conjugation, which indicates that the plasmid might be compatible with all these *Enterobacteriaceae* host species. Besides, these plasmids share a common anti-restriction system that ensures they would not be destroyed by the restriction-modified system in other host strains. Specifically, the pAF18_2 contains an active transposase system with complete IS elements which had acquired the *bla*_CTX-M-3_ gene and an arsenical resistant system. Many other DNA manipulating enzymes, such as integrase and DNA invertase, were also identified in the plasmid, all of which could facilitate the plasmid in efficiently acquiring and transferring antibiotic-resistance genes and other stress-adaptive genes among *Enterobacteriaceae* strains. Unfortunately, due to constraints related to the outbreak of the 2019 novel coronavirus, we were unable to perform conjugation experiments.

### Growth of AF18 in co-cultures and its transcriptional regulation

**Fig. 4:**
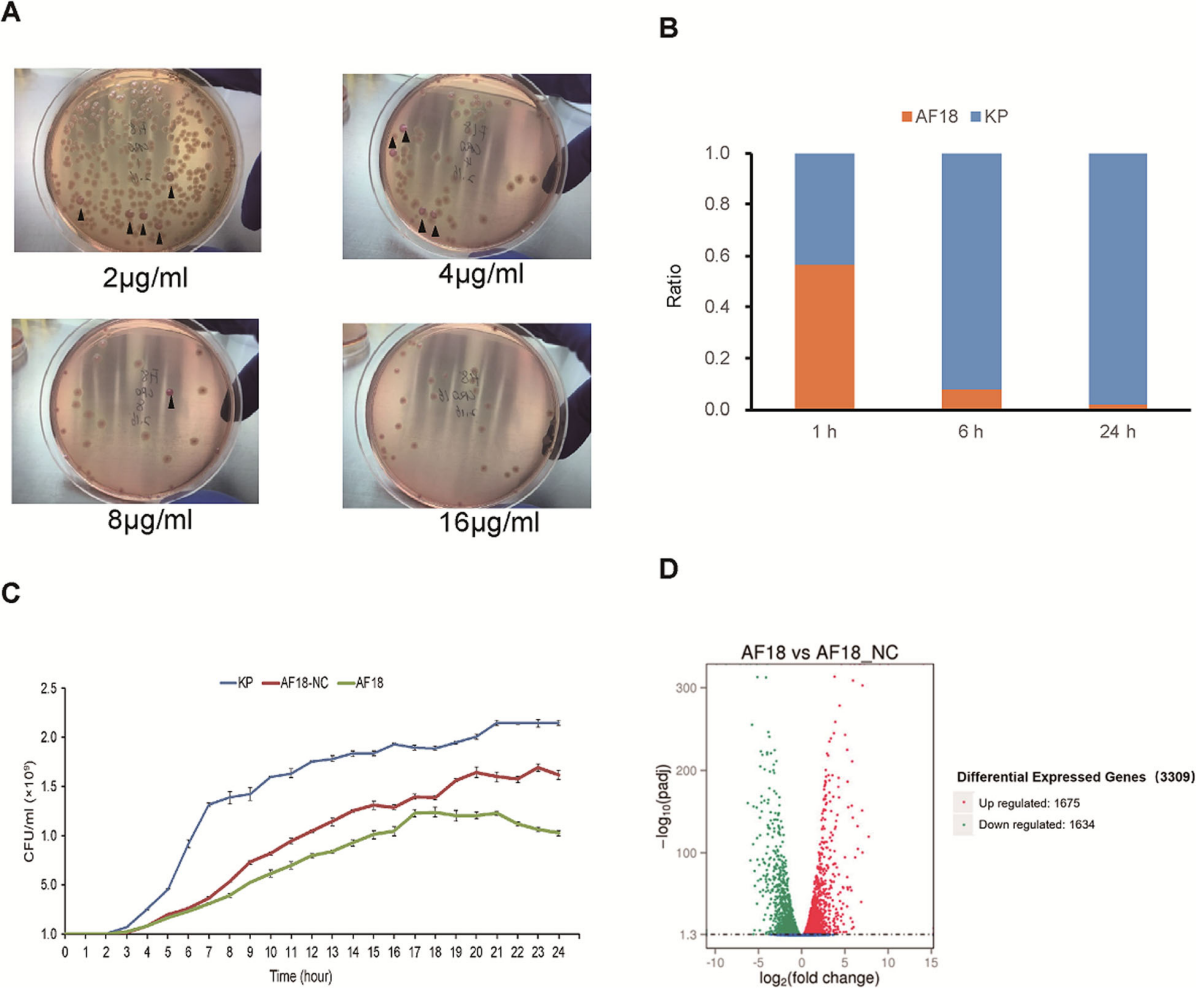
The properties and regulation of the growth rate of AF18. **a** Over-night co-culture of AF18 and the co-infected K. pneumoniae strain in LB medium was spread on MacConkey agar plates supplemented with ceftriaxone at a concentration of 2–16 μg/mL. (▲) stands for *K. pneumoniae* colonies. **b** Proportion of AF18 in the co-culture with the co-infected *K. pneumoniae* strain in LB medium without antibiotic pressure. **c** The growth curves of AF18, AF18-NC and the K. pneumoniae strain. **d** Up- and down-regulated genes in AF18 when compared to the transcriptome of AF18-NC

To disentangle the respective contribution of AF18 and the sensitive *K. pneumoniae* in the co-infection, we co-cultivated the two strain in various concentration of ceftriaxone, and found that addition of 1% of AF18 was able to elevate the MIC from 0.125 μg/ml of pure *K. pneumoniae* culture to 64 μg/ml. Furthermore, when spreading the co-culture onto the MacConkey agar containing ceftriaxone, the sensitive *K. pneumoniae* colonies were able to withstand 8 μg/ml ceftriaxone (Fig. 4a), indicating a strong protective effect of AF18 to the co-infected *K. pneumoniae*.

Although necessary in the co-infection for antibiotic-resistance, AF18 only took less than 1% in the initial sample. Even when equally input, the proportion of AF18 decreased to 1% of the co-culture if without antibiotic pressure (Fig. 4b). It seems that AF18 may be less aggressive, and its growth rate is much slower than the co-inhabited *K. pneumoniae*. It has been reported that plasmid carriage may slow down growth rate due to the cellular cost imposed [19], and thus we generate a new strain—AF18-NC by deleting the resistant plasmid of AF18. Then we measured the independent growth curve of the three strains— *K. pneumoniae*, AF18, and AF18-NC, respectively (Fig. 4c). As expected, AF18-NC did grow faster than its mother strain AF18 since it was relieved from the plasmid-caused cellular cost. However, the growth rate of AF18-NC was still much slower than that of *K. pneumoniae*, suggesting that slow growth is an inherent property of the novel species.

Next, we analyzed the genes involved in the regulation of the growth rate by a comparison between the transcriptomes of AF18 and AF18-NC. A total of 3309 genes of chromosomal coding genes were significantly differentially expressed, with 1675 upregulated and 1634 downregulated in AF18 (Fig. 4d). Functional cluster analysis with GO database showed that most of the differentially expressed genes were in the categories of transcriptional regulation, biosynthesis regulation, metabolic process regulation, signal transduction, and flagellar motility (Fig. S1). Analysis of the non-coding sRNA expression profile identified a total of 15 sRNAs differentially expressed between AF18 and AF18-NC. Interestingly, two of the down-regulated sRNAs in AF18, namely sRNA00063 and sRNA00291 (Fig. S2), shared 98% of their predicted target genes which constitute up to 56% of those differentially expressed genes as mentioned above, suggesting that these two sRNAs have a key role in promoting growth. This result indicated the importance of the two sRNAs in global regulation of growth rate, and consequently, the contribution of the host AF18 in co-infections.

## Discussion

In this study, we reported a case of co-infection caused by a typical pathogen and a rare opportunistic pathogen with taxonomical nomenclature undefined. *K. pneumoniae* is a common pathogen that can cause various aggressive infections [20], while the AF18 strain and its relatives have not been reported to cause infection in humans. In the pathogenic consortium of the co-infection in our study, although taking a very small proportion, AF18 provides strong protection for the entire pathogenic consortium against antibiotic damage. The cooperation between the *K. pneumoniae* and AF18 makes the infectious situation more complicated and difficult in terms of therapies than infections caused by either of them. Meanwhile, as the strain AF18 only took a minor proportion and closely adhered to co-infected *K. pneumoniae*, it was prone to be concealed by the dominant *K. pneumoniae* and hard to be detected and isolated, which led to inaccurate etiological diagnosis and improper anti-infective treatment at first admission. As AF18 and other strains of the same species are rare opportunistic pathogen with little documentation, and conventional testings for bacterial identification are not always correct for such novel species, as shown in this study, WGS comprised a straightforward approach for accurate taxonomy identification.

Sequencing strategy combining both long- and short-read platforms makes it easy to obtain high-quality complete genome and plasmids, which will be helpful for the overall characterization of novel species and deep insight into the functions of the genes they harbor. Phylogenetic analysis with the whole genome of AF18 assigned it to the Enterobacteriaceae family. However, the nomenclature of this novel species is still under discussion due to very limited documentation [21]. The first report of the novel species was in 2016 when *[Kluyvera] intestini* str. GT-16 was isolated from the stomach of a patient with gastric cancer [22], and in the following years, strains of this species were emergingly discovered [23, 24]. Of note, AF18 is the first clear report of human infection of this novel species, as *[Kluyvera] intestine* GT-16 and *Matakosakonia sp.* MRY16_398 were more likely a common resident in the gastrointestinal tract or a by-stander of the diverticulitis. Although the first strain of *[Kluyvera] intestini* str. GT-16 had been assigned to the genus *Kluyvera*, the ANI of strains in this novel species to typical *Kluyvera* spp. are less than 80.8%, even farther than the distance to other genera, such as *Kasokonia* (ANI, 82.3%), and typical *Enterobacter* spp. (ANI, 81%), suggesting that AF18 and its species is not a typical *Kluyvera* species or should not be included in this genus*.* Except AF18 which cause a co-infection with a typical pathogen, two of the other strains of this species (*[Kluyvera] intestini* str. GT-16 [25], and *Matakosakonia sp.* MRY16_398 [23]) were both isolated from patients in Japan, while the other three strains (*Enterobacter sp.* NFR05, *Enterobacter sp.* Bisph2, and *Enterobacteriaceae bacterium* ENNIH2) were isolated from rhizoplane (China), sandy soil (Algeria) [26], and hospital sewage (USA) [24], respectively, indicating a wide range of environments that this species can inhabit.

16S rDNA sequencing is also a commonly used method for species identification; however, there are some matters to consider when using 16S rDNA to determine the taxonomy of a new bacteria. Briefly speaking, we need to obtain the entire 16S rDNA gene (preferably all copies of the strain) and an up-to-date database to ensure accurate identification [18]. Another issue that requires attention is that we indeed need some online websites and databases to help us identify bacterial taxonomy and obtain genomic characteristics of pathogens quickly and accurately. An easy-to-use and convenient web service platform, such as BacWGSTdb (http://bacdb.org/BacWGSTdb) [27], can provide uniform classification criteria to help us identify the taxonomy of species, ARGs, and virulence genes. Moreover, worldwide species information can be integrated into the platform, helping us to evaluate the tracking and evolution of pathogens.

Annotation of genes in the genome supports that AF18 possesses many common features to the *Enterobacteriaceae* family, such as the flagella that confer mobility to the bacterium, many genetic mobile elements that facilitate the transfer of stress-adaptive genes, especially ARGs. In fact, strains of this species have been identified as an important source of extended-spectrum β-lactamase-encoding genes and even carbapenem-resistant genes. For example, *Enterobacteriaceae bacterium* ENNIH2 is KPC-2 positive [24], *Metakosakonia sp.* MRY16–398 carries *bla*_IMP-6_, *bla*_CTX-M-2_, and *aadA2* gene [23], and the *[Kluyvera] intestini* str. GT-16 contains many ARGs that even confer resistance to polymyxin [25]. A more distant relative of AF18—*Pytobacter ursingii* (previously named *Kluyvera intermedia*) was found to be KPC positive and carbapenem-resistant [28]. AF18 carries genes of the *hcp-clp* and *mprAB* system which are powerful in implementing persistence status and endows resistance to many environmental stresses including all kinds of antibiotics [29, 30]. These species can acquire various ARGs from their *Enterobacteriaceae* relatives in natural environments and host gut. For example, *E. coli* and *K. pneumoniae* can share these ARGs through various genetic mobile elements or even in a more efficient manner of conjugating resistant plasmids [31]. Thus, the species of AF18 may prove important in spreading ARGs and function as a mediator in the eco-evolutionary feedback loops of AMR. In this regard, the novel species and many other emerging opportunistic pathogens of *Enterobacteriaceae* family, such as *Kluyvera* spp. and *Enterobacter* spp., deserve more attention in clinical practice and in the field of antibiotic resistance control.

In our study, the resistant AF18 does not have to transfer its resistance gene to the co-infected sensitive *K. pneumoniae* to confer protection. Being antibiotic-resistant by itself, we speculate that AF18 just upregulated the production of antibiotic-hydrolase to generate a niche of low antibiotic concentration for the sensitive *K. pneumoniae* to hide. Such co-operation between different bacteria has been deeply investigated in experiments, and relevant theories or mathematic models have been fully developed which illustrated the important role of growth regulation in maximizing the benefit and population of the entire consortium [32–34]. Our study provides empirical evidence for these hypotheses and highlights the importance of mutualistic relationships between microbes in a co-infection scenario in clinical settings. Transcriptome analysis further identified genes involved in growth regulation and pointed to two novel sRNAs that might be the key regulators of the process. As antibiotics are not always successful, especially in treating opportunistic pathogens, the sRNAs that promote the growth of host strains as we had identified may serve as targets of bacteriostatic agents and deserve further investigations.

## Conclusion

Opportunistic pathogenic strain *Enterobacteriaceae bacterium* AF18 is in a novel species with little documentation. In-depth genomic analysis suggests the ability of this species to transfer between the natural environment and host habitats and in disseminating antibiotic-resistant genes, which potentiates it to be an important disseminator of antibiotic resistance to clinical pathogens. When co-infected with typical pathogens, the resistant opportunistic strain can provide temporary protection for the whole consortium and cause confusions in the etiological diagnosis and antibiotic treatment, although the strain by itself is possibly not a pernicious pathogen for immunocompetent patients. Taken together, *Enterobacteriaceae bacterium* AF18 and other newly emerging opportunistic pathogens complicate the situation of antibiotic resistance control in clinical practice and deserve in-depth investigation including methods for surveillance and control.

## Methods

### Bacterial isolation

The bile sample was collected from a patient with obstructive jaundice who suffered an infection 2 days after the percutaneous transhepatic cholangial drainage (PTCD) surgery admitted to Fujian Province Hospital (N26.08, E119.30) in 2014. The original colonies isolated from the bile sample on a blood agar plate were further incubated in Luria-Bertani (LB) broth overnight. The culture was diluted 10^6^-fold with LB broth and spread onto MacConkey agar and incubated at 37 °C for 24 h. Then, the morphology of the isolated colonies were observed. The *K. pneumoniae* colonies were inoculated into LB broth and incubated overnight. The bacterial cultures were supplemented with glycerol before freezing and stored in a ^− 80^°C freezer. AF18 was selected by culturing overnight in LB broth containing 50 μg/ml ceftriaxone: the aforementioned method was used to pick 10 drug-resistant clones from the MacConkey agar medium; then, the clones were re-inoculated in LB broth containing 50 μg/ml ceftriaxone and cultured overnight. The selection was repeated 5 times, and each culture was supplemented with glycerol before freezing and stored in a ^− 80^°C freezer.

### Biological characterization of strain AF18

#### Gram staining

(1) A total of 10 μl of a bacterial culture that was cultured for 4 h in LB broth without antibiotics was dropped onto a sterile slide. (2) The bacteria were stained for 1 min with ammonium oxalate crystal violet and the dye was washed off with water. (3) A potassium iodide solution was dropped onto the bacteria and allowed to stain for 1 min and the excess dye was gently rinsed off with water. (4) Next, 95% ethanol was dropped onto the slide to destain for 30 s. (5) Safranin solution was dropped onto the slide to counterstain for 30 s, and the dye solution was rinsed off with water. When the slide was dry, the bacteria were observed under a microscope with an oil objective lens.

Biochemical identification: The biochemical identification of AF18 was performed with the VITEK-II automated bacterial identification system and the API20E Enterobacter biochemical identification system (Biomerieux, France). The obtained biochemical properties were compared with the biochemical properties of known species. The VITEK-MS mass spectrometer and the time-of-flight detection method were used to obtain the mass spectrum of AF18, which was compared with the mass spectra of known species. Antimicrobial susceptibility of strain AF18 and *K. pneumonia* was investigated by broth microdilution using the E-test (Biomerieux, France) according to manufacturers’ instructions, and a total of 21 antibiotics were tested as listed in Table 1.

#### Electron microscopy

An inoculation loop was used to scrape a bacterial colony from a blood agar plate. The colony was fixed with 2.5% glutaraldehyde, followed by 1% osmium acid, for 2 h each. After gradient dehydration with 30, 50, 70, 80, 90, and 100% ethanol, the bacteria were dried in a desiccator. After the specimen was coated with gold, the morphology of the bacteria was observed under a Hitachi S-3400 N scanning electron microscope. Bacteria grown on a blood agar plate for approximately 6 h were scraped with an inoculation loop. A bacterial suspension prepared in saline was dropped onto a copper net and allowed to rest for 5 min so that the bacteria could adhere to the copper net; then, the excess water was removed. The specimen was stained with 1% uranyl acetate for approximately 10 min. After the specimen dried, the flagella were observed with a JEM-1230 transmission electron microscope.

### Genome sequencing

AF18 cells were harvest by centrifugation from an overnight culture, and DNA was extracted with QIAamp DNA Mini Kit (Qiagen, Cat No: 51304) following manufacturer’s instruction. The extracted DNA sample was assayed with a NanoDrop spectrophotometer for quantification and then sent to Beijing Novogene Bioinformatics Technology Co., LTD for whole-genome sequencing. The genome of AF18 was both sequenced with the single-molecule real-time sequencing (SMRT) technology from Pacbio using a PacBio RSII sequencer (insert size was approximately 10 kb) for one cell, and sequenced with the short-reads Hiseq 2000 platform from Illumina (100 bp pair-end reads) for 3G raw reads. The obtained raw SMRT reads were analyzed and de novo assembled using SMRT Analysis 2.3.0 software. Then, we performed the error correction of tentative complete circular sequences using Pilon (v1.18) with Illumina short reads.

### Genome annotation

GeneMarks (version 4.17) [35] was used to predict protein-coding genes. The Kyoto Encyclopedia of Genes and Genomes (KEGG), Clusters of Orthologous Groups (COG), NCBI-NR and Gene Ontology (GO) databases were used to annotate the function of predicted genes [36–38]. Virulent genes were reliably identified by BLAST in the Virulence Factors Database (http://www.mgc.ac.cn/VFs/) with identity > 80% and E-value <10e-50, and antibiotic-resistant genes were identified using RGI from the Comprehensive Antibiotic Resistance Database (https://card.mcmaster.ca/) with “perfect and strict hits” and “identity > 90%” [39, 40]. Circos software (Version 0.64) was used to plot a circular map of the genome [41]. Plasmid replicon typing was performed using the curated PlasmidFinder database at the CGE website (https://cge.cbs.dtu.dk/services/PlasmidFinder/) [42].

### Phylogenetic analysis

We used Mash [16] to compare the AF18 genome sequence to the NCBI genome assembly database, and picked 33 genomes from the non-redundant species with identity scores > 75%. Then, we used kSNP3 to identify core genome SNPs (single nucleotide polymorphisms) of each pair of the 34 genome sequences with an optimal k-mer size of 21 (determined by Kchooser) [43]. These core SNPs were used to build a maximum likelihood tree by FastTreeML [44], and iTOL was used to exhibit the phylogenetic tree (https://itol.embl.de/) [45]. We used fastANI to calculated the pairwise ANI of the 34 genome sequences [17]. We used RNAmmer to predict the rRNA sequences [46], and then used BLASTN to align the 16S rRNA sequences to the genomes we picked.

### Comparative analysis of pAF18_2

We used BLASTN to compare pAF18_2 sequence against the NR/NT database and picked 10 non-redundant plasmids according to the query coverage (> 60%) and percent identity (> 99.7%) [47]. We then performed BLASTN for pAF18_2 against the above selected 10 plasmids to find out the alignment fragments with E-value < e-50 [48], and generated the comparative map using CGView (http://wishart.biology.ualberta.ca/cgview/) [49].

### Drug-resistant plasmid elimination test

A single colony of AF18 was inoculated into LB broth medium without antibiotics and cultured for 24 h at 37 °C. Then the culture was 1:1000 diluted and re-inoculated in LB broth medium for another 24 h. The procedure was repeated while an aliquot was collected and spread on LB agar medium for each round of re-inoculation. Colonies grown on the LB agar medium were randomly selected and tested for the presence of *bla*_CTX-M-3_ gene by PCR. DNA was extracted with QIAamp DNA Mini Kit, and PCR was performed under cycling conditions: 95 °C for 5 min, followed by 32 cycles of 95 °C for 1 min, 55 °C for 1 min, 72 °C for 1 min, followed by a single step of 72 °C for 5 min. The pair of Primers were: F 5′-CAGAATAAGGAATCCCATG-3′, and R 5′-CGTCTAAGGCGATAAACA-3′. The PCR negative colonies, which might have lost their resistant plasmid pAF18–2, were functionally confirmed by inoculation in LB broth medium both with and without ceftriaxone (20 μg/ml). The strains that didn’t survive in the ceftriaxone-containing medium were believed to have lost the resistant plasmid, and one of them was preserved and named AF18-NC.

### Co-culture of AF18 and *K. pneumoniae*

AF18 and *K. pneumoniae* were first co-cultured in LB broth mediums containing different concentrations of ceftriaxone (0.125–16 μg/mL) overnight, and then the bacterial solution in the tube was diluted and spread on MacConkey agar medium. We picked out the colonies of *K. pneumoniae* from the plates and incubated them in LB broth medium containing ceftriaxone at the same concentration overnight, and then diluted the bacterial culture with LB broth and spread onto MacConkey agar medium.

### Growth rate measurement

Overnight cultures of the *K. pneumonia* strain, AF18, and AF18-NC were sampled and diluted to O.D. = 0.10, and were then cultured at 37 °C for 24 h in LB broth medium. The turbidity (O.D. value) of the cultures was measured by using a bacterial turbidimeter at 1 h intervals for 24 h, and the 3 bacterial growth curves were drawn based on the O.D. values. The proportion of AF18 and the *K. pneumoniae* strain in co-cultured samples was determined by colony counting on plates of MacConkey agar.

### Transcriptome sequencing

AF18 and AF18-NC cells were harvested from its overnight LB broth medium culture by centrifugation, and total RNA of both strains were extracted with QIAGEN RNeasy Plus Mini Kit (Qiagen, Cat No.74134) following manufacturer’s instruction. The extracted RNA samples were assayed with NanoDrop spectrophotometer for quantification and then sent to Beijing Novogene Co., LTD for transcriptome sequencing. rRNA was removed using the Ribo-Zero rRNA Removal Kit (Epicentre Biotechnologies), then the transcriptome was sequenced on Hiseq 2000 platform with 1G raw reads (paired-end, 2 × 100 bp). Low-quality reads and adaptor sequences were then removed. Using the whole genome of AF18 as the reference genome, the gene expression level for each transcript was estimated by calculating the FPKM (Fragments Per Kilobase per Million mapped fragments) value of each transcript.

### Differential gene expression analysis

The read count data of each transcript was first normalized using DEseq [50]. According to the binomial distribution model, hypothesis testing was performed on each transcript between the AF18 strain and the AF18-NC strain, and confirmed by multiple hypothesis tests.

### Functions of differentially expressed genes

Functions of the differentially expressed genes between AF18 and AF18-NC were annotated with GO database [36], and the probability of enrichment for each cluster was calculated by using the weight algorithm and Fisher’s exact test implemented in topGO package [51]. Clusters with a corrected *p* value < 0.05 were regarded as significantly enriched.

### sRNA analysis

Rockhopper software was used to search new intergenic transcripts [52, 53], and those transcripts that had no hits in the NCBI-NR protein database by BLASTx were considered as candidates for non-coding sRNA. The sRNAs with two-fold increased/decreased FPKM value were regarded as up−/down-regulated. Secondary structures of candidate sRNAs were predicted using RNAfold software and their target genes were predicted by using IntaRNA [54, 55].

## Supplementary information

**Additional file 1:** **Figure S1.** The bar plot of enriched GOs in AF18 and AF18-NC

**Additional file 2:** **Figure S2.** The sequences and the secondary structures of sRNAs

**Additional file 3:** **Table S1.** The results of biochemical testing of AF18 isolate by VITEK II2

**Additional file 4:** **Table S2.** Accession numbers of genome and plasmid sequences used for comparative analyses

**Additional file 5:** **Table S3.** Comparison of whole-genome ANI and 16S rDNA BLASTN results

**Additional file 6:** **Table S4.** The ARGs and virulence genes annotation results of AF18

**Additional file 7:** **Table S5.** The summary statistics of genomic features of AF18

## Data Availability

The complete, annotated genomic sequence of AF18 was deposited in a public database GenBank [56] (accession numbers: chromosome, CP025982; pAF18_1, CP025983; pAF18_2, CP025984). The clean sequence data for RNA-Seq have been deposited in the Genome Sequence Archive [57] in BIG Data Center [58], Beijing Institute of Genomics (BIG), Chinese Academy of Sciences, under accession number CRA002037 that are publicly accessible at https://bigd.big.ac.cn/gsa. Other genome and plasmid sequences used for comparative analyses in this study are publicly available in the NCBI Assembly database (https://www.ncbi.nlm.nih.gov/assembly) and Nucleotide database (https://www.ncbi.nlm.nih.gov/nuccore/) under accession numbers listed in Table S2.

## Abbreviations

AMR: Antimicrobial resistance
ARGs: Antibiotic resistance genes
ANI: Average nucleotide identity
COG: Clusters of Orthologous Groups
FPKM: Fragments Per Kilobase per Million mapped fragments
GO: Gene Ontology
KEGG: Kyoto Encyclopedia of Genes and Genomes
LB: Luria-Bertani
NCBI: National Center for Biotechnology Information
PTCD: Percutaneous transhepatic cholangial drainage
SMRT: Single-molecule real-time sequencing
SNPs: Single nucleotide polymorphisms
