# Supplementary figures and images for: Genomic characterization of an emerging Enterobacteriaceae species: the first case of co-infection with a typical pathogen in a human patient
Source: BMC Genomics. 2020 Apr 15;21:297. doi: 10.1186/s12864-020-6720-z (PMC7156906; doi:10.1186/s12864-020-6720-z)

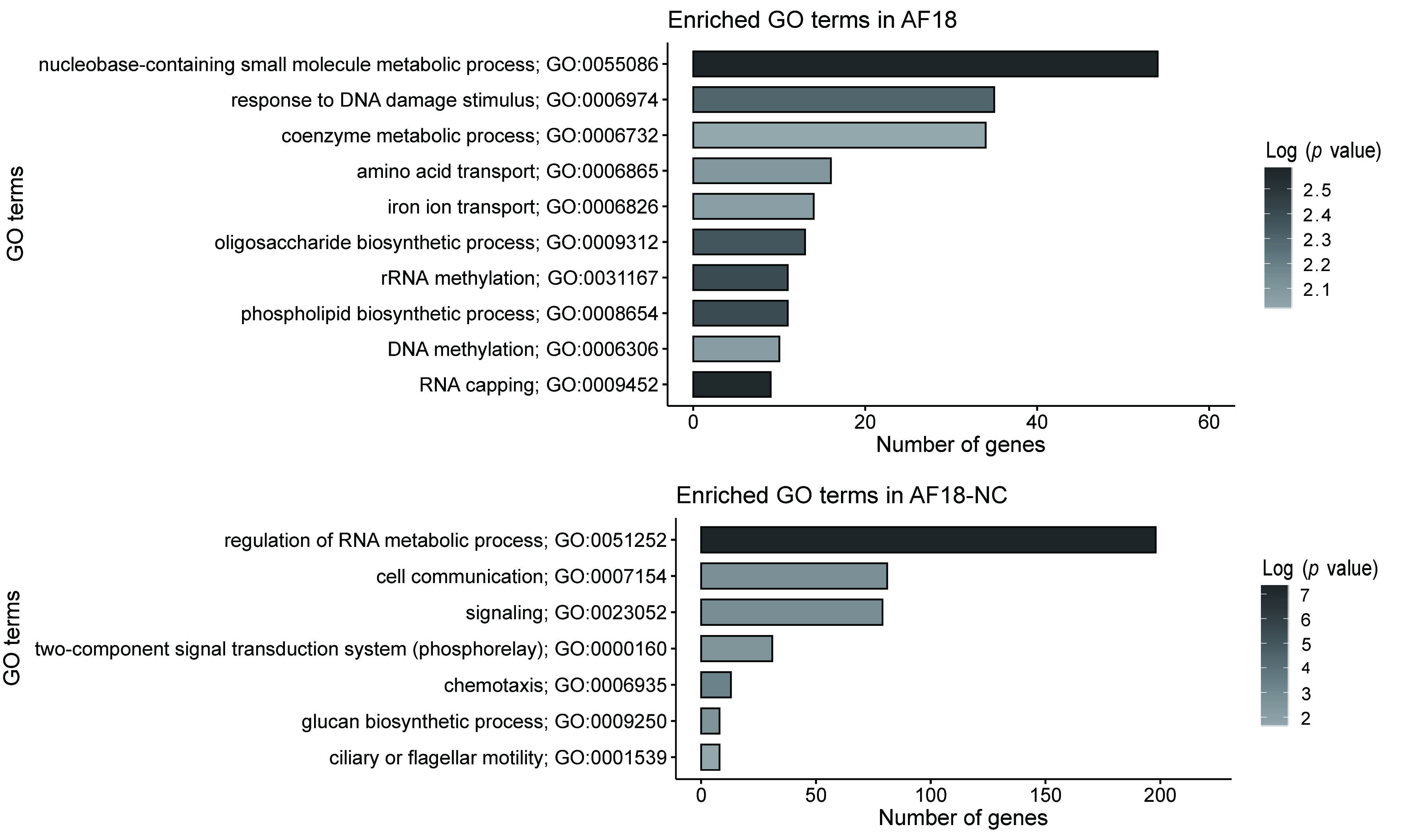

Supplement: Supplementary file 1 — Additional file 1: Figure S1. The bar plot of enriched GOs in AF18 and AF18-NC [file 12864_2020_6720_MOESM1_ESM.tif]

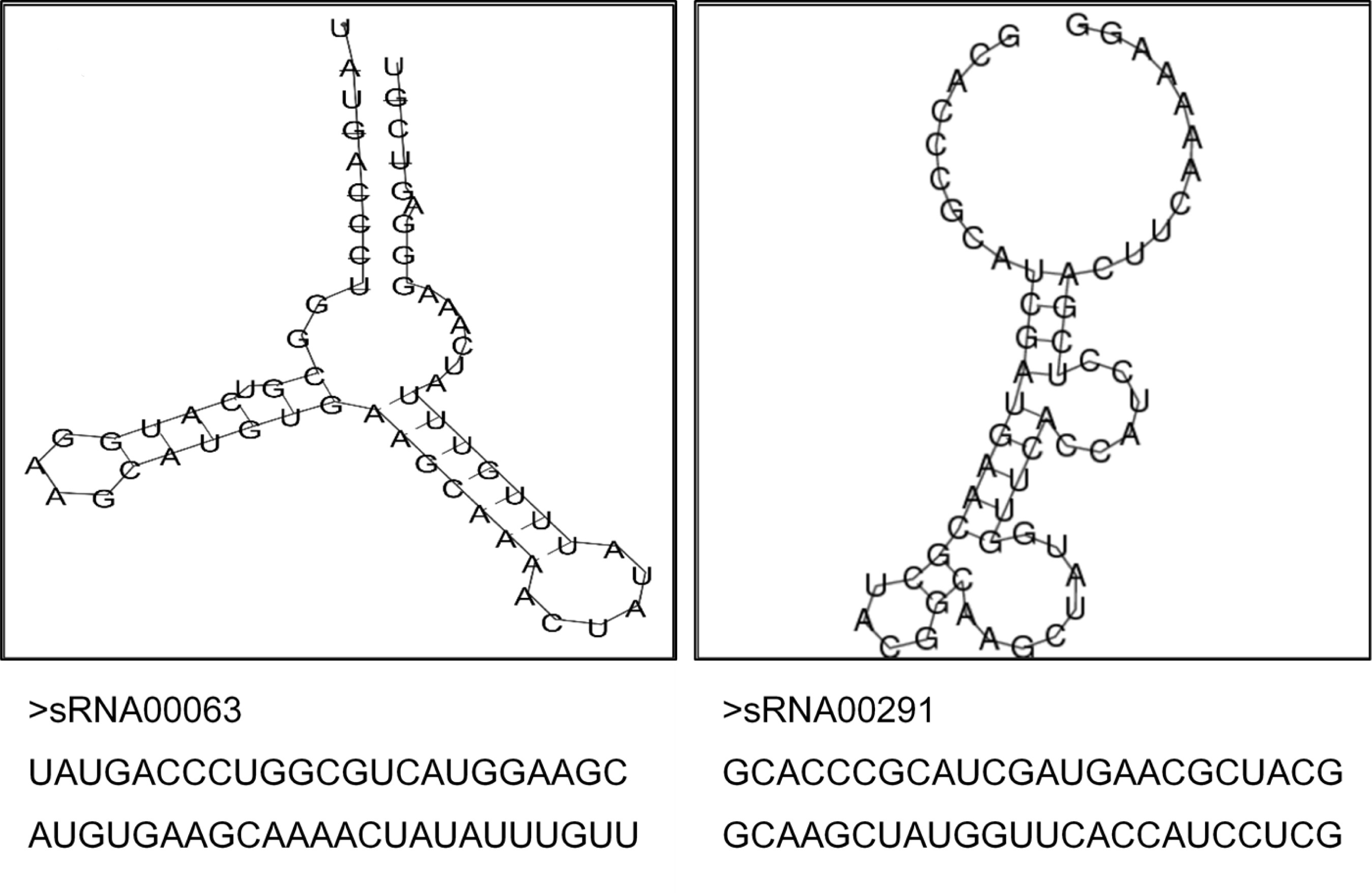

Supplement: Supplementary file 2 — Additional file 2: Figure S2. The sequences and the secondary structures of sRNAs [file 12864_2020_6720_MOESM2_ESM.jpg]
