# Supplementary material for: Genomic characterization of an emerging Enterobacteriaceae species: the first case of co-infection with a typical pathogen in a human patient
Source: BMC Genomics. 2020 Apr 15;21:297. doi: 10.1186/s12864-020-6720-z (PMC7156906; doi:10.1186/s12864-020-6720-z)
Supplement: Supplementary file 3 — Additional file 3: Table S1. The results of biochemical testing of AF18 isolate by VITEK II2 [file 12864_2020_6720_MOESM3_ESM.docx]

Table S1. The biochemical identification results of AF18 isolate by Vitek II.

| Reactions | | Result |
| --- | --- | --- |
| APPA | Ala-Phe-Pro-ARYLAMIDASE | - |
| ADO | ADONITOL | - |
| Pyra | L-Pyrrolydonyl-ARYLAMIDASE | - |
| IARL | L-ARABITOL | - |
| dCEL | D-CELLOBIOSE | + |
| BGAL | BETA-GALACTOSIDASE | + |
| H2S | H2S PRODUCTION | - |
| BNAG | BETA-N-ACETYL-GLUCOSAMINIDASE | - |
| AGLTp | Glutamyl Arylamidase pNA | - |
| dGLU | D-GLUCOSE | + |
| GGT | GAMMA-GLUTAMYL-TRANSFERASE | - |
| OFF | FERMENTATION/GLUCOSE | - |
| BGLU | BETA-GLUCOSIDASE | + |
| dMAL | D-MALTOSE | + |
| dMAN | D-MANNITOL | + |
| dMNE | D-MANNOSE | + |
| BXYL | BETA-XYLOSIDASE | + |
| Balap | BETA-Alanine arylamidase pNA | - |
| ProA | L-Proline ARYLAMIDASE | - |
| LIP | LIPASE | - |
| PLE | PALATINOSE | + |
| TyrA | Tyrosine ARYLAMIDASE | + |
| URE | UREASE | - |
| dSOR | D-SORBITOL | + |
| SAC | SACCHAROSE/SUCROSE | + |
| dTAG | D-TAGATOSE | - |
| dTRE | D-TREHALOSE | + |
| CIT | CITRATE(SODIUM) | + |
| MNT | MALONATE | + |
| 5KG | 5-KETO-D-GLUCONATE | - |
| lLATk | L-LACTATE alkalinisation | + |
| AGLU | ALPHA-GLUCOSIDASE | + |
| SUCT | SUCCINATE alkalinisation | + |
| NAGA | BETA-N-ACETYL-GALACTOSAMINIDASE | - |
| AGAL | ALPHA-GALACTOSIDASE | - |
| PHOS | PHOSPHATASE | - |
| GlyA | Glycine ARYLAMIDASE | - |
| ODC | ORNITHINE DECARBOXYLASE | - |
| LDC | LYSINA DECARBOXYLASE | - |
| lHISa | L-HISTIDINE assimilation | - |
| CMT | COURMARATE | + |
| BGUR | BETA-GLUCORONIDASE | - |
| O129R | o/129 RESISTANCE(comp.vebrio.) | + |
| GGAA | Glu-Gly-Arg-ARYLAMIDASE | - |
| lMLTa | L-MALATE assimilation | - |
| ELLM | ELLMAN | + |
| lLATa | L-LACTATE assimilation | - |
