# Supplementary material for: Genomic characterization of an emerging Enterobacteriaceae species: the first case of co-infection with a typical pathogen in a human patient
Source: BMC Genomics. 2020 Apr 15;21:297. doi: 10.1186/s12864-020-6720-z (PMC7156906; doi:10.1186/s12864-020-6720-z)
Supplement: Supplementary file 4 — Additional file 4: Table S2. Accession numbers of genome and plasmid sequences used for comparative analyses [file 12864_2020_6720_MOESM4_ESM.docx]

| **Table S2 Accession numbers of genome and plasmid sequences used for comparative analyses.** |  |
| --- | --- |
| **Strains** | **GenBank assembly**  **accession number** |
| *[Kluyvera] intestini* strain GT-16 | GCA_001856865.3 |
| *Citrobacter amalonaticus* Y19 | GCA_000981805.1 |
| *Citrobacter farmeri* GTC 1319 | GCA_000764735.1 |
| *Citrobacter portucalensis* strain P10159 | GCA_001281005.1 |
| *Citrobacter rodentium* NBRC 105723 | GCA_000759815.1 |
| *Citrobacter* sp. BIDMC108 | GCA_001037585.1 |
| *Enterobacter cancerogenus* YZ1 | GCA_000478345.1 |
| *Enterobacter cloacae* complex sp. 35734 | GCA_000807415.4 |
| *Enterobacter hormaechei* subsp oharae DSM 16687 | GCA_001729705.1 |
| *Enterobacter kobei* strain GN02825 | GCA_001022915.1 |
| *Enterobacter ludwigii* A3203 | GCA_002022405.1 |
| *Enterobacter* sp. Bisph2 | GCA_000814915.1 |
| *Enterobacter* sp. FY 07 | GCA_001582075.1 |
| *Enterobacter* sp. MGH 8 | GCA_000474805.1 |
| *Enterobacter* sp. NFR05 | GCA_900168315.1 |
| *Enterobacteriaceae bacterium* ENNIH1 | GCA_002903065.1 |
| *Enterobacteriaceae bacterium* ENNIH2 | GCA_002903045.1 |
| *Escherichia coli* PCN061 | GCA_001029125.1 |
| *Klebsiella aerogenes* MGH 62 | GCA_000692175.1 |
| *Klebsiella pneumoniae* BIDMC 41 | GCA_000492195.1 |
| *Kluyvera ascorbata* ATCC 33433 | GCA_000735365.1 |
| *Kluyvera cryocrescens* NBRC 102467 | GCA_001571285.1 |
| *Kluyvera georgiana* ATCC 51603 | GCA_001654985.1 |
| *Kluyvera intermedia* NBRC 102594 | GCA_001598315.1 |
| *Kosakonia oryzae* D4 | GCA_900168185.1 |
| *Kosakonia radicincitans* YD4 | GCA_000877295.1 |
| *Kosakonia sacchari* SP1 | GCA_000300455.4 |
| *Metakosakonia massiliensis* JC163 | GCA_000321045.2 |
| *Metakosakonia* sp. MRY16 398 | GCA_003925915.1 |
| *Pantoea* sp. PSNIH1 | GCA_000784875.3 |
| *Pantoea vagans* 848 PVA | GCA_001067555.1 |
| *Phytobacter ursingii* strain CAV1151 | GCA_001022135.1 |
| *Salmonella enterica* BCW 4232 | GCA_002058415.1 |
| **Plasmids** | **GenBank**  **accession number** |
| *Metakosakonia* sp., pMRY16-398 | AP018758.1 |
| *Escherichia coli*, pECN580 | KF914891.1 |
| *Escherichia coli*, pKPC-LKEc | KC788405.1 |
| *Klebsiella pneumoniae*, pCRKP-1-KPC | KX928750.1 |
| *Escherichia coli* E41-1, p3 | CP028486.1 |
| *Klebsiella oxytoca*, pKPC-8bc0 | CP026277.1 |
| *Morganella morganii*, pMR3-OXA181 | KM660724.1 |
| *Klebsiella pneumoniae*, pKPC-224e | CP026179.1 |
| *Enterobacteriaceae bacterium*, pKPC-c606 | CP026198.1 |
| *Citrobacter freundii*, pIMP-HK1500 | KT989599.1 |
