# Supplementary material for: Genomic characterization of an emerging Enterobacteriaceae species: the first case of co-infection with a typical pathogen in a human patient
Source: BMC Genomics. 2020 Apr 15;21:297. doi: 10.1186/s12864-020-6720-z (PMC7156906; doi:10.1186/s12864-020-6720-z)
Supplement: Supplementary file 5 — Additional file 5: Table S3. Comparison of whole-genome ANI and 16S rDNA BLASTN results [file 12864_2020_6720_MOESM5_ESM.docx]

**Table S3 Comparison of whole-genome ANI and 16S rDNA BLASTN results**

| **Strain A** | **Strain B** | **ANI** | **Average identity of 7 copies of 16S rDNA** |
| --- | --- | --- | --- |
| AF18 | *Kluyvera [intestini]* strain GT-16 | 98.80 | 99.72 |
| AF18 | *Metakosakonia sp* MRY16-398 | 98.78 | 99.85 |
| AF18 | *Enterobacteriaceae bacterium* ENNIH2 | 98.58 | 99.85 |
| AF18 | *Enterobacteriaceae bacterium* ENNIH1 | 92.54 | 99.03 |
| AF18 | *Phytobacter ursingii* strain CAV1151 | 92.24 | 99.09 |
| AF18 | *Metakosakonia massiliensis* JC163 | 83.16 | 97.72 |
| AF18 | *Kosakonia sacchari* SP1 | 82.32 | 97.70 |
| AF18 | *Kosakonia oryzae* D4 | 82.29 | 97.82 |
| AF18 | *Kosakonia radicincitans* YD4 | 82.23 | 97.72 |
| AF18 | *Enterobacter sp* FY-07 | 82.08 | 97.66 |
| AF18 | *Enterobacter cancerogenus* YZ1 | 81.57 | 97.38 |
| AF18 | *Enterobacter hormaechei* subsp oharae DSM 16687 | 81.54 | 97.43 |
| AF18 | *Enterobacter cloacae* complex sp 35734 | 81.49 | 97.00 |
| AF18 | *Citrobacter portucalensis* strain P10159 | 81.07 | 97.67 |
| AF18 | *Enterobacter kobei* strain GN02825 | 81.01 | 97.24 |
| AF18 | *Citrobacter amalonaticus* Y19 | 80.76 | 97.86 |
| AF18 | *Citrobacter rodentium* NBRC 105723 | 80.69 | 97.43 |
| AF18 | *Citrobacter sp* BIDMC108 | 80.67 | 97.48 |
| AF18 | *Citrobacter farmeri* GTC 1319 | 80.61 | 97.94 |
| AF18 | *Klebsiella aerogenes* MGH-62 | 80.57 | 97.46 |
| AF18 | *Kluyvera ascorbata* ATCC 33433 | 80.56 | 97.37 |
| AF18 | *Escherichia coli* PCN061 | 80.51 | 97.13 |
| AF18 | *Salmonella enterica* BCW_4232 | 80.42 | 97.42 |
| AF18 | *Klebsiella pneumoniae* BIDMC 41 | 80.39 | 97.86 |
| AF18 | *Escherichia coli* BIDMC 19A adjGK | 80.31 | 96.73 |
| AF18 | *Pantoea sp* PSNIH1 | 77.60 | 95.85 |
| AF18 | *Pantoea vagans* 848_PVA | 77.56 | 96.55 |
