# Supplementary material for: Genomic characterization of an emerging Enterobacteriaceae species: the first case of co-infection with a typical pathogen in a human patient
Source: BMC Genomics. 2020 Apr 15;21:297. doi: 10.1186/s12864-020-6720-z (PMC7156906; doi:10.1186/s12864-020-6720-z)
Supplement: Supplementary file 7 — Additional file 7: Table S5. The summary statistics of genomic features of AF18 [file 12864_2020_6720_MOESM7_ESM.docx]

**Table S5. The summary statistics of genomic features of AF18**

|  |  |
| --- | --- |
| **Attribute** | **Value** |
| Length of coding DNA (bp) | 5,110,764 bp |
| Gene length/Genome (%) | 0.8722 |
| Coding genes | 5885 |
| Genes with function prediction | 5758 |
| Genes assigned to NR | 5496 |
| Genes assigned to GO | 4001 |
| Genes assigned to COGs | 4468 |
| Genes assigned to KEGG | 3553 |
| Genes assigned to VFDB | 74 |
| Genes assigned to ARDB | 15 |
| ncRNA genes | 178 |
| Genomic islands | 20 |
| Prophage | 11 |
| CRISPR | 5 |
